# Pharmacokinetic Drug Interaction Study of Sorafenib and Morphine in Rats

**DOI:** 10.3390/pharmaceutics13122172

**Published:** 2021-12-16

**Authors:** Agnieszka Karbownik, Danuta Szkutnik-Fiedler, Tomasz Grabowski, Anna Wolc, Joanna Stanisławiak-Rudowicz, Radosław Jaźwiec, Edmund Grześkowiak, Edyta Szałek

**Affiliations:** 1 Department of Clinical Pharmacy and Biopharmacy, Poznań University of Medical Sciences, 14 Św. Marii Magdaleny Str., 61-861 Poznań, Poland; akarbownik@ump.edu.pl (A.K.); stanisl@interia.pl (J.S.-R.); grzesko@ump.edu.pl (E.G.); eszalek@ump.edu.pl (E.S.); 2 Preclinical Development, Polpharma Biologics SA, Trzy Lipy 3, 80-172 Gdańsk, Poland; tomasz.grabowski@polpharmabiologics.com; 3 Department of Animal Science, Iowa State University, 239E Kildee Hall, Ames, IA 50011, USA; awolc@iastate.edu; 4 Research and Development, Hy-Line International, 2583 240th Street, Dallas Center, IA 50063, USA; 5 Department of Gynecological Oncology, University Hospital of Lord’s Transfiguration, Poznań University of Medical Sciences, 84/86 Szamarzewskiego Str., 60-101 Poznań, Poland; 6 Laboratory of Mass Spectrometry, Institute of Biochemistry and Biophysics PAS, Polish Academy of Sciences, 5A Pawińskiego Str., 02-106 Warsaw, Poland; rjazwiec@ibb.waw.pl

**Keywords:** pharmacokinetics, drug–drug interaction, sorafenib, sorafenib N-oxide, morphine

## Abstract

A combination of the tyrosine kinase inhibitor—sorafenib—and the opioid analgesic—morphine—can be found in the treatment of cancer patients. Since both are substrates of P-glycoprotein (P-gp), and sorafenib is also an inhibitor of P-gp, their co-administration may affect their pharmacokinetics, and thus the safety and efficacy of cancer therapy. Therefore, the aim of this study was to evaluate the potential pharmacokinetic drug–drug interactions between sorafenib and morphine using an animal model. The rats were divided into three groups that Received: sorafenib and morphine (I_SOR+MF_), sorafenib (II_SOR_), and morphine (III_MF_). Morphine caused a significant increase in maximum plasma concentrations (C_max_) and the area under the plasma concentration–time curves (AUC_0–t_, and AUC_0–∞_) of sorafenib by 108.3 (*p* = 0.003), 55.9 (*p* = 0.0115), and 62.7% (*p* = 0.0115), respectively. Also, the C_max_ and AUC_0–t_ of its active metabolite—sorafenib N-oxide—was significantly increased in the presence of morphine (*p* = 0.0022 and *p* = 0.0268, respectively). Sorafenib, in turn, caused a significant increase in the C_max_ of morphine (by 0.5-fold, *p* = 0.0018). Moreover, in the presence of sorafenib the C_max_, AUC_0–t_, and AUC_0–∞_ of the morphine metabolite M3G increased by 112.62 (*p* < 0.0001), 46.82 (*p* = 0.0124), and 46.78% (*p* = 0.0121), respectively. Observed changes in sorafenib and morphine may be of clinical significance. The increased exposure to both drugs may improve the response to therapy in cancer patients, but on the other hand, increase the risk of adverse effects.

## 1. Introduction

About 50% of cancer patients undergoing active anti-cancer therapy and 90% of patients in the advanced stages experience severe pain [1]. In the clinical practice of cancer therapies, it can be expected that a combination of tyrosine kinase inhibitors (TKIs), such as sorafenib, and opioid analgesics, including morphine, are used, which, according to the WHO guidelines, is the first-choice opioid in the relief of cancer pain [2].

Sorafenib belongs to the group of multi-kinase inhibitors, showing antiproliferative activity of cancer cells and anti-angiogenic properties [3]. Molecularly targeted therapy, an example of which is sorafenib, is the most promising form of pharmacotherapy among cancer patients. The use of this drug is indicated in the treatment of hepatocellular carcinoma (HCC), which is one of the most common cancers worldwide and is the third most common cause of cancer death [4]. Sorafenib is also used in patients with advanced renal cell carcinoma (RCC) [5], of which 30% of patients are in metastatic state at diagnosis, as well as in the treatment of differentiated thyroid cancer, including metastatic cancer, in which the use of radioactive iodine is ineffective [6].

Sorafenib is mainly metabolized in the liver, where it undergoes N-oxidation with CYP3A4 to become the pharmacologically active sorafenib N-oxide (SR_NO), and then is transformed to inactive glucuronides as a result of UDP-glucuronosyltransferase (UGT1A9 and UGT1A1) enzyme activity [7]. The pharmacokinetics of sorafenib and its metabolites depend on many efflux transporters, such as organic cation transporter−1 (OCT1), organic anion transporting polypeptide 1B1/3 (OATP1B1/3), multidrug resistance-associated protein 2 (MRP2) and protein 3 (MRP3), breast cancer resistance protein (BCRP), and P-glycoprotein (P-pg), which is also an inhibitor [8,9]. Sorafenib has been shown to inhibit P-gp in vitro [3,10] and in vivo [11,12]. After simultaneous administration of sorafenib and P-gp substrates, such as digoxin thereof, the growth of their plasma concentrations cannot be excluded [3,10].

Morphine is metabolized primarily by the UGT enzyme, which is expressed in the liver, small intestine, brain, and other organs [13]. In humans, the glucuronidation of morphine is mediated via a UGT2B7 isoform in the liver, and the major metabolite of morphine is morphine-3-glucuronide (M3G), which doesn’t have analgesic properties and may antagonize the pharmacological effects of morphine [13,14,15]. Only 10% of morphine is metabolized to morphine-6-glucuronide (M6G), which has a greater analgesic activity than morphine [16]. Both M3G and M6G are highly hydrophilic, but only M6G can penetrate the blood-brain barrier (BBB) because M3G does not bind to μ-, δ- and κ-opioid receptors [15]. UGT1A1, 1A3, and 1A9 are also involved in the metabolism of morphine but to a lesser extent [16].

Some in vitro [17,18,19], in vivo [17,20,21,22,23,24,25], and clinical [26,27,28] studies have shown that morphine is probably a substrate of P-gp. Likewise, its glucuronidated metabolites M3G [29] and M6G [30] are considered to be substrates of this drug transporter [31]. Animal studies have shown that P-gp inhibitors increase central nervous system (CNS) concentrations and antinociceptive effects of morphine [21,22,23,24,31]. A significantly increased morphine analgesia [32] and brain distribution [20,31,33] were observed in P-gp-deficient mice, suggesting that its analgesic effect is considerably dependent on P-gp expression. A study by Hamabe et al. [34] showed a statistically significant negative correlation between the analgesic effect of morphine and the expression of P-gp in the cerebral cortex. Lötsch et al. showed that inhibition of P-gp in rats caused an increased central nervous system uptake and enhanced antinociception of M6G [30]. Clinical studies also support the hypothesis that morphine and its glucuronide metabolites are P-gp substrates [26,27,29,31]. Fudin et al. [26] showed that the P-gp inducer rifampin decreased morphine blood concentrations, and that the P-gp inhibitor telaprevir [35] may increase intestinal morphine absorption. It has been proven that patients with P-gp positive tumors require a higher dose of morphine to achieve an analgesic effect [27].

However, there are also numerous reports that neither morphine [29,36] nor M3G [37] and M6G [29,31,37] are P-gp substrates. Both in vitro [38] and clinical [39] studies suggest that morphine appears to be a likely OCT1 substrate/inhibitor [31,38] and that MRP2 and MRP3 are involved in the hepatic excretion of M3G and M6G [31,40]. Bourasset et al. [41] indicated in turn that M6G is not a P-gp or MRP1 substrate, but rather a GLUT1 and/or a weak OATP2-like substrate at the BBB in mice. So, the effect of P-gp on the pharmacokinetics of morphine is not yet elucidated.

Bearing in mind that sorafenib and probably morphine belong to the P-gp substrates, and sorafenib is also a P-gp inhibitor [10,11,12], the combined use of these drugs may alter their pharmacokinetics, and finally, the safety and efficacy of the cancer therapy. This drug combination in cancer patients may be associated then with drug–drug interactions (DDI) with a higher risk of treatment disruption [42].

Therefore, the aim of this study was to evaluate the potential pharmacokinetic DDIs between sorafenib and morphine using an animal model. The results may be helpful in clinical practice, as they can contribute to determining the appropriate pharmacotherapy in cancer patients.

## 2. Materials and Methods

Adult, healthy, fed male Wistar rats (*n* = 23) at the age of 14 weeks were used in the study. Animals were given a standard diet, and free access to fresh water was provided. All required guidelines (international, national, and/or institutional) for the care and use of animals were followed. The study was performed according to a protocol approved by the Local Ethics Committee (consent No. 36/2018 from 20 July 2018), Poznań University of Life Sciences, Department of Animal Physiology and Biochemistry, Wołyńska 35 Str., 60-637 Poznań, Poland.

### 2.1. Animals

All animals were divided into three groups: one study group (I_SOR+MF_)—rats received sorafenib with morphine (*n* = 8, weight 491.88 ± 7.99 g), and two control groups (II_SOR_ and III_MF_)—rats received sorafenib (*n* = 8, weight 502.75 ± 16.42 g) or morphine (*n* = 7, weight 491.43 ± 8.52 g), respectively, along with 1 mL of the 10% dimethyl sulfoxide (DMSO) for sorafenib and 0.9% sodium chloride for morphine.

The body weight of the animals did not differ significantly between the groups.

Sorafenib (100 mg/kg body weight (b.w.) [43]; Nexavar^®^, batch number BXHT61, Bayer AG, Leverkusen, Germany) was administrated directly into the stomach of animals (I_SOR+MF_ and II_SOR_) using a gastric probe (1 mL of each solution). The 10% DMSO was used to prepare the sorafenib tosylate solution. An aqueous solution of morphine (5 mg/kg b.w. [13]; Morphine Sulfas WZF, 20 mg/mL, batch number O1DR1118, Warszawskie Zakłady Farmaceutyczne Polfa S.A., Warsaw, Poland) was administered into the peritoneal cavity of animals (I_SOR+MF_ and III_MF_).

100 µL of blood was collected from each rat by cutting off a piece of his tail. Blood samples were collected before drug administration and at the following time points after administration: 0.5, 1, 2, 3, 4, 5, 6, 7, 8, 10, 12, 24, 30, 48, 72, and 96 h. The plasma was separated by centrifugation at 2880× *g* for 10 min at 4 °C and stored at −80 °C until drug analysis.

### 2.2. Reagents

The following reagents were used during the HPLC-UV (high-performance liquid chromatography with ultraviolet detection) and UPLC-MS/MS (ultra-high performance liquid chromatography tandem mass spectrometry) assays: acetonitrile (CAS number 75-05-8), ammonium acetate (CAS 631-61-8), ethyl acetate (CAS 141-78-6), lapatinib (CAS 231277-92-9), methanol (CAS 67-56-1) purchased from Sigma-Aldrich (Saint Louis, MO, USA); glacial acetic acid (CAS 64-19-7), formic acid (CAS 64-18-6), morphine D6 solution (CAS 67-56-1), ultrapure water (deionized, distilled and filtered through Direct Q3 system) (CAS 7732-18-5) purchased from Merck Millipore (Burlington, MA, USA); sorafenib (CAS 284461-73-0), sorafenib N-oxide (CAS 583840-03), morphine (CAS 57-27-2), morphine-3-glucuronide (CAS 20290-09-9) purchased from LGC (Teddington TW11 0LY, UK).

### 2.3. HPLC-UV Assay of Sorafenib and SR_NO

The HPLC-UV method was used to determine the blood plasma concentrations of sorafenib and its metabolite SR_NO. Analyzes were carried out using the HPLC Waters 2695 Separations Module with an autosampler and the Waters 2487 Dual Absorbance Detector [43,44]. The supernatant volume for each time point used to prepare the HPLC sample was 20 μL.

An ammonium acetate 0.1 M, pH = 3.4 (eluent A) and acetonitrile (eluent B) (1:1, *v*/*v*) solution were used as a mobile phase. The flow rate was set at 1.0 mL/min. The linear gradient ran from 60% eluent A and 40% eluent B to 29% eluent A and 71% eluent B. Other HPLC parameters were as follows: the temperature of the column (a reversed-phase Symmetry^®^ C8, 250 × 4.6 mm, 5.0 μm particle size, Waters Corporation^®^, Milford, MA, USA) was 25 °C, the UV detection wavelength was 265 nm, the injection volume was 20 μL. Lapatinib was used as the internal standard (IS). The calibration curves were linear within the range of 0.025−3.0 µg/mL (R^2^ = 0.999), and 0.025−0.40 µg/mL (R^2^ = 0.999), for sorafenib and SR_NO respectively. The high intra- and inter-day precision (coefficients of variation, CV < 13%) and accuracy (%bias < 14%) for sorafenib, and SR_NO were obtained. The lower limit of quantification (LLOQ) was 0.025 µg/mL for both sorafenib and SR_NO.

### 2.4. UPLC-MS/MS Assay of Morphine and M3G

Plasma morphine and M3G concentrations were assayed using ultra-performance liquid chromatography-tandem mass spectrometry (UPLC-MS/MS). The instrumentation used was a Waters Xevo TQ-MS mass spectrometer with a standard electrospray ion source coupled to a Waters Acquity UPLC. The calibration curve for morphine consisted of 6 samples in the range from 1 to 500 ng/mL (R^2^ = 0.999), and for M3G it consisted of 6 samples in the range from 2 to 1000 ng/mL (R^2^ = 0.995).

As the internal standard (IS), a solution of morphine D6 in methanol in the concentration of 20 ng/mL was used. Every sample was injected into the instrument in the amount of 20 μL. Chromatographic separation was done on a Waters Acquity HSS C18, with a 1.8 μm, 2.1 × 50 mm analytical column at a temperature of 70 °C. The 1.5 min gradient method was used. The mobile phases were: A—0.1% formic acid in MQ and B—0.1% formic acid in methanol. The gradient changed from 7% B to 80% B in 0.8 min by a slightly convex curve. At 1.0 min, it returned to the starting condition for column equilibration. Using the flow rate of 1 mL/min, the obtained retention time of morphine and morphine D6 was 0.47 min, and M3G was 0.23 min. The parameters of the mass spectrometer were as follows: positive polarity mode; the capillary voltage—2.5 kV, desolvation temperature—650 °C, desolvation gas flow—1000 L/h.

Morphine was analyzed using two transmissions 286.14→155.09 (Collision energy 35) and 286.14→165.07 (CE 40); MorD6 292.18→165.02 (CE 35), and M3G 462.18→286.14 (CE 30).

### 2.5. Pharmacokinetic Evaluation

The following pharmacokinetic parameters of sorafenib, SR_NO, morphine, and M3G were calculated using the Phoenix^®^ WinNonlin version 8.0 software (Certara, Princeton, NJ, USA): the elimination rate constant (k_e_), the absorption rate constant (k_a_), the half-life in the elimination phase (t_1/2_), the area under the concentration–time curve from zero to the last measurable concentration (AUC_0–t_), the area under the plasma concentration–time curve from zero to infinity (AUC_0–∞_), the apparent plasma drug clearance (Cl/F), and the apparent volume of distribution (V_d_/F).

The maximum plasma concentration (C_max_) and the time to reach the C_max_ (t_max_) were obtained directly from the measured values.

All of the abovementioned parameters underwent statistical analysis.

Dose/kg-adjusted drug concentrations were calculated according to the following equations: drug concentration (µg/mL for sorafenib and ng/mL for morphine)/drug dose (mg) and drug concentration (µg/mL for sorafenib and ng/mL for morphine)/drug dose per body mass kilogram (mg/kg).

### 2.6. Statistical Analysis

The statistical analysis was performed using SAS software, version 9.4 (SAS Institute Inc., Cary, NC 27513, USA). The Shapiro-Wilk test was used to determine the normality. Two pairs of groups were analyzed: I_SOR+MF_ vs. II_SOR_ and I_SOR+MF_ vs. III_MF_ independently. The differences between the normally distributed variables were determined with the Student’s t-test. Not-normally distributed variables were analyzed using the Kruskal-Wallis test, and a *p*-value of <0.05 was considered significant.

## 3. Results

All the data were expressed as the arithmetic mean value ± standard deviation (SD).

### 3.1. The Influence of Morphine on the Pharmacokinetics of Sorafenib and SR_NO

**Figure 1:**
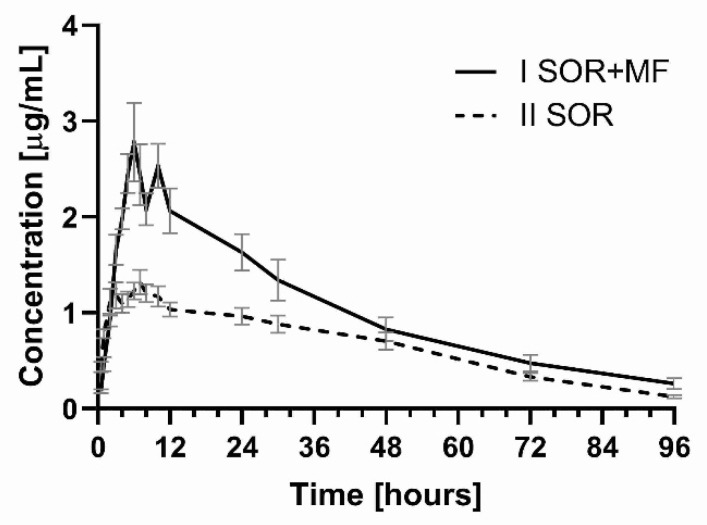
The sorafenib plasma concentration–time profiles in rats receiving sorafenib (II_SOR_) and sorafenib + morphine (I_SOR+MF_).

When sorafenib was co-administered with morphine (I_SOR+MF_ group), the C_max_, AUC_0–t_, and AUC_0–∞_ of sorafenib significantly increased by 108.3 (*p* = 0.003), 55.9 (*p* = 0.0115), and 62.7% (*p* = 0.0115), respectively (Table 1, Figure 1). Significantly lower k_a_ (by 95.9%, *p* = 0.0008) and CL/F (by 36.3%, 0.0224) values were observed for sorafenib in the group of rats receiving both drugs when compared to the sorafenib treatment group alone (II_SOR_ group). However, the k_el_ of sorafenib in the I_SOR+MF_ group was significantly higher (by 7.3-fold, *p* = 0.0008). No statistically significant differences were revealed for t_max_, t_1/2_, and V_d_/F when compared to the II_SOR_ group (Table 1).

**Figure 2:**
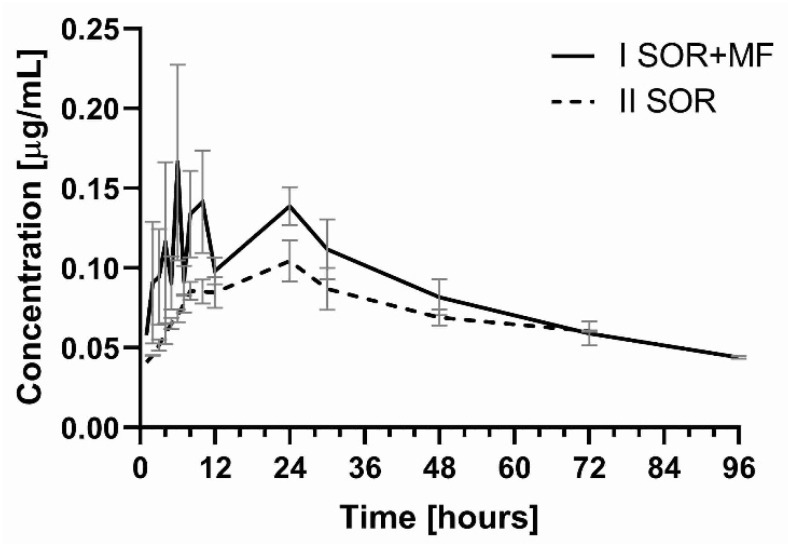
The sorafenib N-oxide plasma concentration–time profiles in rats receiving sorafenib (II_SOR_) and sorafenib + morphine (I_SOR+MF_).

In the presence of morphine, statistically higher values of C_max_ (by 1.5-fold, *p* = 0.0022) and AUC_0–t_ (by 0.6-fold, *p* = 0.0268) for SR_NO were observed (Table 1, Figure 2). There were no significant differences among the groups for the following pharmacokinetic parameters of SR_NO: AUC_0−∞_, t_max_, k_el_, and t_1/2_ (Table 1).

The sum of the C_max_ for sorafenib and SR_NO values was also significantly higher (*p* < 0.001; G_mean_ ratio = 2.11, and 90% CI = 1.75; 2.54) in the group treated with morphine (3.52 ± 0.75 µg/mL; CV = 21.3) than the II_SOR_ group (1.68 ± 0.37 µg/mL; CV = 21.8).

The C_max_, AUC_0–t_, and AUC_0–__∞_ SR_NO/sorafenib ratios were similar in both groups (Table 1).

### 3.2. The Influence of Sorafenib on the Pharmacokinetics of Morphine and M3G

**Figure 3:**
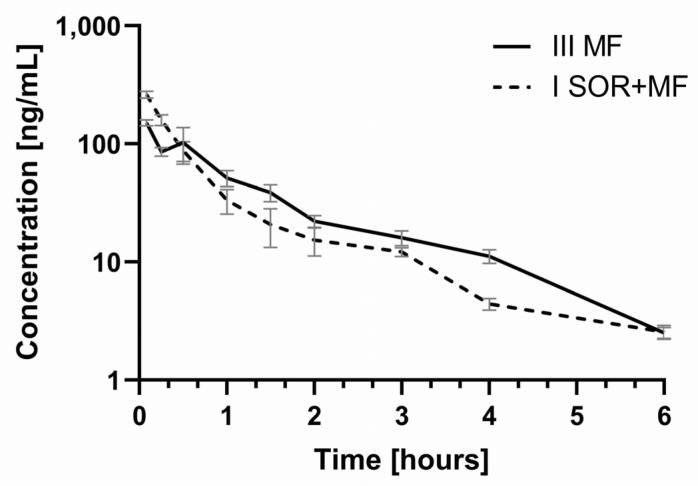
The morphine plasma concentration–time profiles in rats receiving morphine (III_MF_) and sorafenib + morphine (I_SOR+MF_).

After the administration of morphine with sorafenib, the C_max_ of morphine increased by 0.5-fold (*p* = 0.0018) when compared with the morphine treatment group (Table 2, Figure 3). However, no statistically significant differences were revealed for the AUC_0–t_ and AUC_0–∞_ of morphine. There were no significant differences among the groups for the following pharmacokinetic parameters of morphine: t_max_, k_el_, t_1/2_, Cl, and V_d_/F (Table 2).

**Figure 4:**
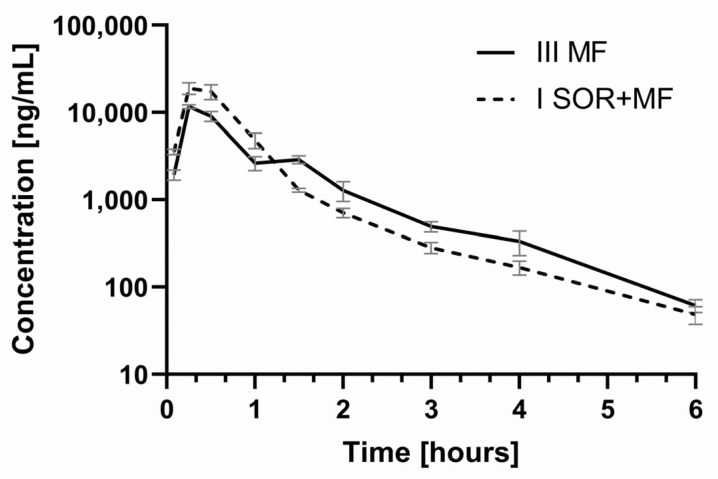
The morphine-3-glucuronide plasma concentration–time profiles in rats receiving morphine (III_MF_) and sorafenib + morphine (I_SOR+MF_).

When morphine and sorafenib were co-administered, the exposure to M3G was significantly higher when compared with the morphine treatment group (Table 2, Figure 4). In the presence of sorafenib, the C_max_, AUC_0–t_, and AUC_0–∞_ of M3G increased by 112.62 (*p* < 0.0001), 46.82 (*p* = 0.0124), and 46.78% (*p* = 0.0121), respectively. No statistically significant differences were revealed for the t_max_, k_el_, and t_1/2_ of M3G (Table 2).

The ratios of the C_max_, AUC_0–t_, and AUC_0–∞_ of M3G/morphine in comparison to the III_MF_ group were increased by 28.67 (*p* = 0.2004), 51.05 (*p* = 0.0488), and 47.58% (*p* = 0.0435), respectively.

## 4. Discussion

We evaluated the pharmacokinetics of two P-gp substrates—sorafenib (which is also a potent P-gp inhibitor) and morphine (for which the role of the P-gp in the pharmacokinetics is unclear), which were co-administered in healthy rats.

The doses of sorafenib (100 mg/kg b.w., orally) and morphine (5 mg/kg b.w., intraperitoneally) used in our study were selected based on the available literature [12,13] and did not cause any adverse effects in healthy rats. The study is not free of limitations. The pharmacokinetic profile of sorafenib and morphine may be different between Wistar rats and humans, and we did not measure the concentrations of sorafenib glucuronide. No animals that modelled HCC or induced pain were used. However, using healthy animals allowed for eliminating the impact of comorbidities on the inter-individual variability.

### 4.1. The Influence of Morphine on the Pharmacokinetics of Sorafenib and SR_NO

Pharmacokinetic interactions of practical importance are mainly based on changes in drug absorption and metabolism [42,45,46]. Our study showed that the co-administration of morphine and sorafenib resulted in a higher exposure of sorafenib when compared to sorafenib alone. It was documented by an approx. 1.1-fold higher C_max_ (the ratio C_max_ I_SOR+MF_/C_max_ II_SOR_ was 2.08) and higher values of AUC_0–t_ and AUC_0–∞_ (by 0.6-fold) in the I_SOR+MF_ group when compared to the control animals (II_SOR_ group). Apart from that, a significant decrease in the k_a_ (0.03 vs. 0.74 h^−1^) was noted in the I_SOR+MF_ group. This fact may be the reason for the observed increase in the C_max_ of sorafenib in the presence of morphine (Table 1, Figure 1).

A similar tendency was observed for the main active metabolite of sorafenib—SR_NO, where C_max_ and AUC_0–t_ also increased in the I_SOR+MF_ group (by 145.5 and 61.9%, respectively) (Table 1, Figure 2). Also, the sum of the C_max_ for sorafenib and SR_NO values was significantly higher (*p* < 0.001) in the group treated with morphine than the II_SOR_ group.

Since SR_NO shows an in vitro potency similar to that of sorafenib, the risk of toxicity is further enhanced. A higher sorafenib N-oxide exposure probably follows an increased exposure to sorafenib. However, the ratios of sorafenib N-oxide/sorafenib for the C_max_, AUC_0–t_, and AUC_0–∞_ in the II_SOR_ and I_SOR+MF_ group did not differ significantly, demonstrating no effect of morphine on sorafenib oxidation.

According to the FDA guidelines [47], the significantly highest C_max_ value is generally related to the toxicity of a drug and is used therefore to evaluate or predict the risk of toxicity changes in DDI studies. In the evaluation of DDIs by regulatory agencies, systemic exposure is based on the plasma AUC_0–t_. Following FDA [47] and EMA [48] classification, the AUC_0–t_ change is considered to influence the drug’s efficacy but also its toxicity. Those guidelines indicate that an AUC_0–t_ ratio (AUC_0–t_ in the presence of a perpetrator/AUC_0–t_ in the absence of a perpetrator) ≥5, 2–5, and 1.25–2 determined a strong, moderate, and weak pharmacokinetic-based DDI occurrence, respectively. In our study, the AUC_0–t_ of sorafenib in the presence of morphine was significantly higher, but the ratio of AUC_0–t_ I_SOR+MF_/AUC_0–t_ II_SOR_ was 1.56.

Considering the above, in clinical practice, the increased systemic exposure of sorafenib and its active N-oxide metabolite in the presence of morphine may improve the response to therapy, but on the other hand, the higher C_max_ could be associated with the severity of such adverse events as gastrointestinal disorders or hand-foot syndrome [3,10].

A significant role of P-gp on the absorption of tyrosine kinase inhibitors has been studied [42,49]. Sorafenib, which is similar to, for example, critozinib and lapatinib, is a P-gp substrate, the absorption of which may depend on this efflux transporter [42]. Co-administration of another substrate or inhibitor of P-gp might lead then to a clinically relevant DDI in the absorption phase, such as reducing the absorption rate constant observed in our study. Sorafenib t_max_ values may confirm more prolonged sorafenib absorption in the I_SOR+MF_ group when compared to the II_SOR_ group (7.63 vs. 5.13 h), although these differences were not statistically significant (*p* = 0.0616). In addition, there was a parallel increase of the AUC_0–t_ of sorafenib and its metabolite SR_NO in the I_SOR+MF_ group when compared to the II_SOR_ group. Moreover, there were no statistically significant differences for the SR_NO/sorafenib ratios for the C_max_, AUC_0–t_ and AUC_0–∞_. This may confirm an interaction of sorafenib and morphine at the absorption level.

We have observed indeed a significantly lower clearance of sorafenib in the I_SOR+MF_ group (by 0.4-fold). Since sorafenib is largely eliminated by hepatic metabolism and excreted in feces as metabolites or unchanged, DDIs caused by changes in renal elimination may not be of significant importance. In addition, the k_el_ of sorafenib was increased in the presence of morphine. However, it cannot be excluded that P-gp present in the kidneys may be of some importance in the renal elimination of all tyrosine kinase inhibitors, including sorafenib [42,50]. However, in the presence of morphine, the CL/F and Vd/F of sorafenib decreased to a similar extent (about 70%) and the t_1/2_ did not increase significantly when compared to the sorafenib alone group, which speaks to the lack of interaction of these drugs at the level of metabolism.

In summary, observed changes in sorafenib pharmacokinetics in the I_SOR+MF_ group may be due to the competition between sorafenib and morphine for their incorporation in the P-gp transport pathway. It could be related to the concentration-dependent inhibitory effects of morphine and M3G on P-gp. This fact may be checked in the continuation of this study, considering multiple administrations of morphine to achieve a steady-state and fluctuation of the opioid concentrations.

### 4.2. The Influence of Sorafenib on the Pharmacokinetics of Morphine and M3G

Since the role of P-gp in the pharmacokinetics of morphine and its glucuronidated metabolites is unclear, we investigated the effect of one of the more potent P-gp inhibitors—sorafenib—on the pharmacokinetics of morphine and its M3G metabolite in rats. Animal studies have shown, for example, a relationship between the use of the other potent P-gp inhibitors—such as elacridar—and the increase in the penetration of drugs across the BBB, e.g., digoxin, talinolol, quinidine, docetaxel, and sunitinib [42,51].

The results of our study showed that the co-administration of morphine with sorafenib caused the increase of the C_max_ of morphine by 56.94% (the ratio C_max_ I_SOR+MF_/C_max_ III_MF_ was 1.57) (Table 2, Figure 3). Thismorphine plasma C_max_ increase in the I_SOR+MF_ group may involve the risk of its adverse effects. The AUC values did not differ significantly between the groups. The ratio AUC_0–t_ I_SOR+MF_/AUC_0–t_ III_MF_ was 0.91, and the confidence intervals (90% CI) were: 0.75; 1.16, and 0.78; 1.17, respectively. This is also confirmed by the calculation of dose-adjusted values (Table 2). It is noteworthy that, in the presence of sorafenib, the plasma C_max_, AUC_0–t_, and AUC_0–∞_ of M3G and the ratios (M3G/morphine) for AUC_0–t_ and AUC_0–∞_ were also significantly higher (Table 2). It is known that inactive M3G may be responsible for neurotoxic symptoms, such as myoclonus or hyperalgesia, as well as allodynia [12,15]. The higher M3G/morphine AUC ratios in the I_SOR+MF_ group in our study may indicate the impact of sorafenib on morphine’s analgesic efficacy and safety. Unfortunately, we did not evaluate the pharmacological effects of morphine, which is a limitation of this study. However, we can suppose that a higher C_max_ of morphine may not cause stronger analgesic activity because it is compensated by a higher concentration of M3G, which reduces the pharmacological effects of morphine. However, we cannot exclude the increased risk of side effects, which M3G causes.

The analgesic efficacy of morphine is mainly associated with its ability to penetrate the BBB barrier, which is firmly dependent on membrane transporters, including brain P-gp. Brain P-gp can mediate the efflux of morphine from the brain, reducing its analgesic effect. Therefore, the level of P-gp expression in the brain is related to the analgesic effects of this opioid, and drugs that are brain P-gp inhibitors may decrease morphine efficacy [27]. There is also documented that chronic morphine exposure induces P-glycoprotein in the rat brain what may enhance morphine efflux from the brain, reducing its analgesic activity [22].

It should also be taken into account that morphine-related adverse reactions and its analgesic efficacy are generally associated with genetics polymorphism [13]. Although, there are divergent research results on this issue as well. Some studies showed that ABCB1 polymorphisms impact morphine PK/PD [23,52], but Coulbault et al. [53] claims it does not affect. Moreover, changes in the pharmacokinetics of this opioid do not necessarily affect the analgesic effect or the severity of side effects [14].

Meissner et al. [28] documented that when a P-gp inhibitor cyclosporine was co-administered with morphine in healthy volunteers, an increase in the AUC value of morphine was noted. Although, the authors suggested that this may not have any significant clinical effects.

We did not achieve statistically significant differences in the AUC_0–t_ and AUC_0–∞_ of morphine between the I_SOR+MF_ and III_MF_ groups. Also, the clearance, t_1/2_, and other pharmacokinetic parameters of morphine were not changed between the groups (Table 2). Therefore, the elevated plasma levels of morphine in the presence of sorafenib are probably not strongly caused by the inhibition of the P-gp efflux transporter in the proximal renal tubule, but rather by P-gp inhibition during the absorption phase. However, decreased renal excretion of M3G cannot be ruled out, because M3G has a higher affinity to renal P-gp than morphine itself, because it is more hydrophilic. Drewe et al. [29] also suggested that P-gp inhibition may have a greater effect on the tubular secretion of M3G than on M6G; therefore P-gp inhibition will more likely increase M3G plasma concentrations.

The increase in plasma M3G after the intravenous administration of morphine in streptozocin-induced rats was observed, among others, by Hasegawa et al. [54]. The fact that inhibition of Pgp in rats does not affect the volume of distribution and clearance of morphine was confirmed by Letrent et al. [21] and Drewe et al. [29]. Drewe et al. [29] found that P-gp inhibition caused an increase in the AUC and t_1/2_ of M3G, while the pharmacokinetics of morphine and its active metabolite M6G were not changed. Gadeyne et al. [14] showed that P-gp inhibitors, ketoconazole, and elacridar did not have significant effects on morphine pharmacokinetics in dogs, although dogs in the ketoconazole group had higher sedation scores.

## 5. Conclusions

Observed changes in the pharmacokinetics of sorafenib and morphine co-administered may be due to competition between the two drugs for their inclusion in the P-gp transport pathway. This fact may be of clinical relevance. Increased exposure to both drugs may improve the response to therapy in cancer patients, but on the other hand, it may increase the risk of their side effects, which should be noted.

## Figures and Tables

**Table 1:** Pharmacokinetic parameters of sorafenib and its metabolite sorafenib N-oxide after a single oral dose of100 mg/kg b.w. of sorafenib (II_SOR_ group) and a single oral dose of 100 mg/kg b.w. of sorafenib + single intraperitoneal dose 5 mg/kg b.w. of morphine (I_SOR+MF_ group).

Pharmacokinetic Parameters	II_SOR_ (*n* = 8)	I_SOR+MF_ (*n* = 8)	*p*-Value I_SOR+MF_ vs. II_SOR_	G_mean_ Ratio * (90% CI) I_SOR+MF_ vs. II_SOR_
sorafenib				
Cmax (µg/mL)	1.56 ± 0.35	3.25 ± 0.80	0.0030	2.08 (1.70; 2.55)
	(22.6)	(24.6)		
Cmax/(D/kg)	0.03 ± 0.01	0.07 ± 0.02	<0.0001	2.13 (1.73; 2.62)
(kg × µg/mL/mg)	(23.9)	(23.6)		
AUC0–t	62.83 ± 16.14	97.98 ± 30.17	0.0115	1.53 (1.16; 2.02)
(µg × h/mL)	(25.7)	(30.8)		
AUC0–t/(D/kg)	1.25 ± 0.32	1.99 ± 0.59	0.0075	1.57 (1.20; 2.05)
(µg × h × kg/mL/mg)	(25.4)	(29.7)		
AUC0–∞ (µg × h/mL)	67.05 ± 16.70	109.08 ± 37.18	0.0115	1.58 (1.17; 2.13)
	(24.9)	(34.1)		
AUC0–∞/(D/kg)	1.33 ± 0.33	2.21 ± 0.73	0.0076	1.62 (1.21; 2.16)
(µg × h × kg/mL/mg)	(24.9)	(32.9)		
t_max_ (h)	5.13 ± 2.17 (42.3)	7.63 ± 2.72 (35.7)	0.0616	1.54 (1.06; 2.25)
k_a_ (h^−1^)	0.74 ± 0.31 (42.5)	0.03 ± 0.01 (20.5)	0.0008	0.03 (0.03; 0.05)
k_el_ (h^−1^)	0.035 ± 0.01 (30.3)	0.29 ± 0.16 (53.5)	0.0008	8.16 (5.98; 11.12)
t_1/2_ (h)	21.89 ± 7.79 (35.6)	27.31 ± 5.32 (19.5)	0.0829	1.25 (1.02; 1.53)
Cl/F (L/h × kg)	0.80 ± 0.22 (27.1)	0.51 ± 0.23 (43.8)	0.0224	0.62 (0.46; 0.83)
V_d_/F (L)	25.30 ± 11.59 (45.8)	19.14 ± 5.26 (27.5)	0.0829	0.77 (0.59; 1.01)
sorafenib N-oxide				
C_max_ (µg/mL)	0.11 ± 0.02 (21.8)	0.27 ± 0.16 (57.5)	0.0022	2.15 (1.50; 3.08)
AUC_0–t_ (µg × h/mL)	4.10 ± 1.56 (38.1)	6.64 ± 2.44 (36.8)	0.0268	1.64 (1.14; 2.36)
AUC_0–∞_ (µg × h/mL)	8.61 ± 2.19 (25.4)	9.39 ± 2.97 (31.6)	0.1242	1.41 (0.95; 2.08)
t_max_ (h)	16.38 ± 8.21 (50.1)	14.50 ± 11.40 (78.6)	0.4531	0.76 (0.41; 1.39)
k_el_ (h^−1^)	0.016 ± 0.010 (60.9)	0.023 ± 0.012 (51.6)	0.4001	0.80 (0.47; 1.36)
t_1/2_ (h)	53.31 ± 25.23 (47.3)	39.30 ± 22.54 (57.4)	0.4622	1.25 (0.73; 2.13)
ratio sorafenib N-oxide/sorafenib				
C_max_ (µg/mL)	0.07 ± 0.02 (26.8)	0.09 ± 0.07 (76.2)	0.5280	1.03 (0.67; 1.61)
AUC_0–t_ (µg × h/mL)	0.07 ± 0.02 (37.1)	0.07 ± 0.03 (43.7)	0.6012	1.07 (0.70; 1.65)
AUC_0–∞_ (µg × h/mL)	0.14 ± 0.05 (38.1)	0.10 ± 0.07 (64.1)	0.9176	0.89 (0.53; 1.51)

C_max_, maximum observed plasma concentration; AUC_0–t_, area under the plasma concentration-time curve from zero to the last measurable concentration; AUC_0–∞_, area under the plasma concentration-time curve from zero to infinity; t_max_, time to reach the C_max_; k_a_, absorption rate constant; k_el_, elimination rate constant; t_1/2_, half-life in the elimination phase; Cl/F, apparent plasma drug clearance; V_d_/F, apparent volume of distribution; b.w., body weight; the pharmacokinetic parameter values are shown as the arithmetic means ± standard deviations (SD) with coefficients of variation (CV) (%) in the brackets; * geometric means (G_mean_) ratio between I_SOR+MF_ and II_SOR_ groups (%) with a 90% confidence interval (CI) in the brackets; individual drug ratios were calculated according the following equations: the metabolite C_max_ (ng/mL)/parent C_max_ (ng/mL), metabolite AUC_0–t_ (ng × h/mL)/parent drug AUC_0–t_ (ng × h/mL), and metabolite AUC_0–∞_ (ng × h/mL)/parent drug AUC_0–∞_ (ng × h/mL).

**Table 2:** Pharmacokinetic parameters of morphine and its metabolite M3G after a single intraperitoneal dose of 5 mg/kg b.w. of morphine (III_MF_ group) and a single oral dose of 100 mg/kg b.w. of sorafenib + single intraperitoneal dose of 5 mg/kg b.w. of morphine (I_SOR+MF_ group).

Pharmacokinetic Parameters	III_MF_ (*n* = 7)	I_SOR+MF_ (*n* = 8)	*p*-Value I_SOR+MF_ vs. III_MF_	G_mean_ Ratio * (90% CI) I_SOR+MF_ vs. III_MF_
morphine				
Cmax (ng/mL)	166.83 ± 46.12	261.83 ± 47.85	0.0018	1.59 (1.30; 1.94)
	(27.6)	(18.3)		
Cmax/(D/kg)	67.88 ± 18.74	106.14 ± 18.96	0.0018	1.58 (1.30; 1.93)
(kg × ng/mL/mg)	(27.6)	(17.9)		
AUC0–t (ng × h/mL)	169.88 ± 47.37	155.40 ± 28.41	0.4786	0.93 (0.75; 1.16)
	(27.9)	(18.3)		
AUC0–t/(D/kg)	69.28 ± 19.99	63.06 ± 11.52	0.4655	0.93 (0.75; 1.16)
(ng × h × kg/mL/mg)	(28.9)	(18.3)		
AUC0–∞ (ng × h/mL)	174.44 ± 46.73	162.57 ± 25.89	0.5458	0.95 (0.78; 1.17)
	(26.8)	(15.9)		
AUC0–∞/(D/kg)	71.14 ± 19.75	65.96 ± 10.49	0.5287	0.95 (0.77; 1.17)
(ng × h × kg/mL/mg)	(27.8)	(15.9)		
t_max_ (h)	0.14 ± 0.16 (110.2)	0.08 ± 0.00 (0.0)	0.3559	0.77 (0.47; 1.27)
k_el_ (h^−1^)	0.60 ± 0.18 (29.3)	0.54 ± 0.41 (76.1)	0.2976	0.75 (0.46; 1.23)
t_1/2_ (h)	1.22 ± 0.28 (22.7)	1.91 ± 1.13 (59.3)	0.2976	1.33 (0.82; 2.15)
Cl/F (L/h × kg)	15.05 ± 4.39 (29.2)	15.51 ± 2.36 (15.2)	0.8010	1.06 (0.86; 1.30)
V_d_/F (L/kg)	27.49 ± 13.38 (48.7)	44.65 ± 29.20 (65.4)	0.1778	1.40 (0.76; 2.60)
				
M3G				
C_max_ (ng/mL)	9781.28 ± 3184.17 (32.6)	20,796.81 ± 3657.84,(17.6)	<0.0001	2.20 (1.73; 2.80)
AUC_0–t_ (ng × h/mL)	10,035.88 ± 1408.48 (14.0)	14,734.53 ± 3979.45 (27.0)	0.0124	1.43 (1.14; 1.79)
AUC_0–∞_ (ng × h/mL)	10,131.53 ± 1393.15 (13.8)	14,871.32 ± 4001.35 (26.9)	0.0121	1.42 (1.14; 1.79)
t_max_ (h)	0.46 ± 0.09 (20.4)	0.34 ± 0.13 (37.6)	0.0662	0.71 (0.53; 0.96)
k_el_ (h^−1^)	0.73 ± 0.20 (27.6)	0.68 ± 0.19 (28.0)	0.6355	0.93 (0.69; 1.24)
t_1/2_ (h)	1.03 ± 0.33 (32.5)	1.13 ± 0.47 (41.1)	0.7285	1.08 (0.81; 1.45)
	M3G/morphine			
C_max_ (ng/mL)	63.87 ± 30.71 (48.1)	82.18 ± 21.67 (26.4)	0.2004	1.14 (0.78; 1.66)
AUC_0–t_ (ng × h/mL)	64.45 ± 24.06 (37.3)	97.35 ± 33.05 (34.0)	0.0488	1.38 (0.96; 2.00)
AUC_0–∞_ (ng × h/mL)	62.88 ± 22.44 (35.7)	92.80 ± 28.44 (30.7)	0.0435	1.53 (1.10; 2.12)

C_max_, maximum observed plasma concentration; AUC_0–t_, area under the plasma concentration-time curve from zero to the last measurable concentration; AUC_0–∞_, area under the plasma concentration-time curve from zero to infinity; t_max_, time to reach the C_max_; k_a_, absorption rate constant; k_el_, elimination rate constant; t_1/2_, half-life in the elimination phase; Cl/F, apparent plasma drug clearance; V_d_/F, apparent volume of distribution; b.w., body weight; the pharmacokinetic parameter values are shown as the arithmetic means ± standard deviations (SD) with coefficients of variation (CV) (%) in the brackets; * geometric means (G_mean_) ratio between I_SOR+MF_ and II_SOR_ groups (%) with a 90% confidence interval (CI) in the brackets; individual drug ratios were calculated according the following equations: the metabolite C_max_ (ng/mL)/parent C_max_ (ng/mL), metabolite AUC_0–t_ (ng × h/mL)/parent drug AUC_0–t_ (ng × h/mL), and metabolite AUC_0–∞_ (ng × h/mL)/parent drug AUC_0–∞_ (ng × h/mL).

## Data Availability

The data presented in this study are available on request from the corresponding author.
